# Terminal anorexia nervosa is a dangerous term: it cannot, and should not, be defined

**DOI:** 10.1186/s40337-022-00599-6

**Published:** 2022-06-07

**Authors:** Angela S. Guarda, Annette Hanson, Philip Mehler, Patricia Westmoreland

**Affiliations:** 1 Department of Psychiatry and Behavioral Sciences, Johns Hopkins School of Medicine, Baltimore, MD 21287 USA; 2 Department of Psychiatry, University of Maryland, Jessup, MD 20794 USA; 3 Department of Medicine, University of Colorado School of Medicine, Aurora, CO USA; 4 Department of Psychiatry, University of Colorado Anschutz Medical Center, Aurora, CO 80045 USA

**Keywords:** Anorexia nervosa, Terminal anorexia, Medical assistance in dying, Physician assisted suicide, Capacity, Competence, Involuntary treatment, Severe and enduring anorexia nervosa, Physician assisted death, Severe and enduring eating disorders

## Abstract

A recent article (JED 10:23, 2022) proposed defining terminal anorexia to improve access to palliative and hospice care, and to medical aid in dying for a minority of patients with severe and enduring anorexia nervosa (SE-AN). The authors presented three cases and, for two, the first author participated in their death. Anorexia nervosa is a treatable psychiatric condition for which recovery may be uncertain. We are greatly concerned however regarding implications of applying the label “terminal” to anorexia nervosa and the risk it will lead to unjustified deaths in individuals whose mental illness impairs their capacity to make a reasoned treatment decision.

## Introduction

Gaudiani et al., [1] recently proposed preliminary criteria for “terminal anorexia” and describe three illustrative cases. Proposed characteristics include: (1) Age over 30, (2) Prior persistent engagement in high-quality, multidisciplinary eating disorders care and (3) decision-making capacity to understand further treatment is futile, the wish not to prolong life and acceptance of death. They argue defining terminal anorexia will improve access to palliative and hospice care, and to medical assistance in dying (MAID), otherwise known as physician assisted suicide (PAS) for select patients with severe and enduring anorexia nervosa (SE-AN), which itself is poorly defined [2–4]. For one patient, a posthumous co-author, the first author completed MAID forms, and for another prescribed lethal medication. We find the label “terminal anorexia” and application of PAS to anorexia nervosa (AN) deeply troubling.

Experienced clinicians who treat AN inevitably encounter patients who succumb to their illness, as AN has high mortality [5]. Palliative care is increasingly recognized as relevant for a minority of adults with SE-AN who do not respond to expert intensive inpatient behavioral treatment, and in whom continued, aggressive attempts at weight restoration infringe on patient autonomy, risk harm, and may constitute futile care [6, 7]. Palliative care focuses on improving quality of life and on symptom relief rather than cure of the underlying medical condition, however it does not require a terminal diagnosis and can be prescribed separately or conjointly with curative care. The first case presented by the authors appears to meet this standard.

## Terminal anorexia cannot be defined

AN is a treatable psychiatric disorder. Evidence suggests the majority of those affected will eventually recover, although recovery is often protracted occurring after years, and sometimes decades of illness [8]. Risk factors for mortality are poorly defined and there are no clear staging criteria for SE-AN comparable to those used for cancer to help define terminal cases (estimated survival of six months or less). Most individuals with SE-AN who are over the age of 30, remain ill and have previously engaged in high-quality care, will not die within six months. Although the risk of death from starvation in SE-AN is real, even seasoned clinicians cannot predict who will recover or when, nor identify “those who will not be able to survive”, and most medical complications are reversible with nutritional rehabilitation and expert care [9]. Furthermore, malnutrition exacerbates depressive and obsessional symptoms, promotes eating disordered cognitions and impairs decision making [10, 11]. Ambivalence towards treatment, a hallmark symptom of AN, results in treatment avoidance [12]. For adults who have not responded to outpatient care, inpatient treatment in a behavioral specialty program is recommended [13] and achieving a healthy weight in intensive treatment is the strongest predictor of recovery [14–16]. Concerningly, two of the cases described by Gaudiani et al.[1], were offered PAS without ever receiving adequate inpatient specialty care. One patient spent 1–2 weeks in intensive treatment before leaving against medical advice, another was hospitalized for medical stabilization but thereafter declined admission to a residential program. In severe AN, when a patient’s life is at risk, involuntary treatment provided by a behavioral inpatient specialty program, can be lifesaving, and when effective is often met with gratitude by patients [17–20]. When such treatment is inaccessible, or has failed, or when repeated intensive weight restoration is followed by equally rapid weight loss and relapse, other approaches, including harm reduction and palliative care, that focus on improvements in quality of life, can still foster hope in eventual recovery and motivation to reverse malnutrition.

## Decision making capacity regarding treatment in AN is impaired

AN impairs patients’ ability to freely choose to accept the life-saving medicine they need most, food. Gaudiani et al.[1] argue that the cases presented have decisional capacity to refuse care and (in two cases) to consent to PAS. Mental capacity to consent to or refuse a treatment is task specific and requires understanding risks and benefits and weighing the pros and cons of proposed options, appreciating how they apply to one’s own condition and making and communicating a reasoned, consistent choice [21]. Problematically, standardized tests of competence fail to identify many individuals with AN whose capacity is impaired [22, 23] and most general psychiatrists and palliative care specialists have little experience assessing capacity in patients with AN. Assessment of capacity to refuse care in AN is especially challenging as most patients appear rational in all ways but one—their ability to fully appreciate how the risks and benefits of treatment decisions apply to their own condition. How then, can they appreciate the risks and benefits of MAID or PAS? As PAS is neither a curative nor a reversible intervention, we believe competency to consent to MAID should have a high threshold.

Determining capacity to consent to PAS additionally includes a statutory requirement for the assessment of non-coercion, as persuasion and encouragement of a vulnerable individual is coercive. In one case mentioned by Gaudiani et al.[1], PAS was suggested by the treating author, in another case the idea of treatment futility was reinforced despite the patient not completing a full course of intensive treatment.

## The danger of a label

Defining criteria for terminal anorexia has potential risks. We urge extreme caution in discussing these ideas, as vulnerable patients and their caregivers are but a Google search away. Increased public demand for PAS for psychiatric illnesses in Europe following legalization of MAID is a warning [24–26]. Introducing the diagnosis of “terminal anorexia” risks countering hope for recovery and increasing demand for PAS by patients with SE-AN. Like social contagion and suicide, demoralized patients who read about terminal AN, may self-identify with the label and seek out concierge physicians, who offer PAS as an elective “treatment”. Defining terminal anorexia risks colluding with anorectic reasoning that improved quality of life and potential for recovery are hopeless prospects. High disease burden in SE-AN, including physical, social and financial costs can contribute to extreme hopelessness and guilt. Coupled with the driven compulsive nature of the disorder and its inextricable tie to identity [27], the idea of life without AN may seem unimaginable, out of reach. Both patients and their exhausted caregivers, under the guidance of a trusted MAID prescriber, may then view PAS as a logical appealing solution to their suffering, even when they have yet to undertake adequate treatment for their illness.

## Conclusion

We need to develop treatments and combat inequalities in availability and access to care for patients with SE-AN; treatments that nurture hope of recovery, focus on functional rehabilitation and improve quality of life, and that may for some, include palliative care. Defining terminal anorexia inherently conflicts with these goals and we fear will lead to unjustified deaths for a treatable condition.

## Data Availability

Not applicable.

## Abbreviations

AN: Anorexia nervosa
MAID: Medical aid in dying
PAS: Physician assisted suicide
SE-AN: Severe and enduring anorexia nervosa
